# A reflection on *A House in the Sky* by Amanda Lindhout and Sara Corbett

**DOI:** 10.3402/ejpt.v5.25305

**Published:** 2014-10-01

**Authors:** Karestan C. Koenen

**Affiliations:** 1 Department of Epidemiology, Mailman School of Public Health, Columbia University, New York, NY, USA; 2 Past-President, International Society of Traumatic Stress Studies, Deerfield, IL, USA

Review of *A House in the Sky* by Amanda Lindhout and Sara Corbett (2014). New York, United States: Scribner. 400 pp. ISBN 978-1-45165613, 16 USD.

**Figure:**
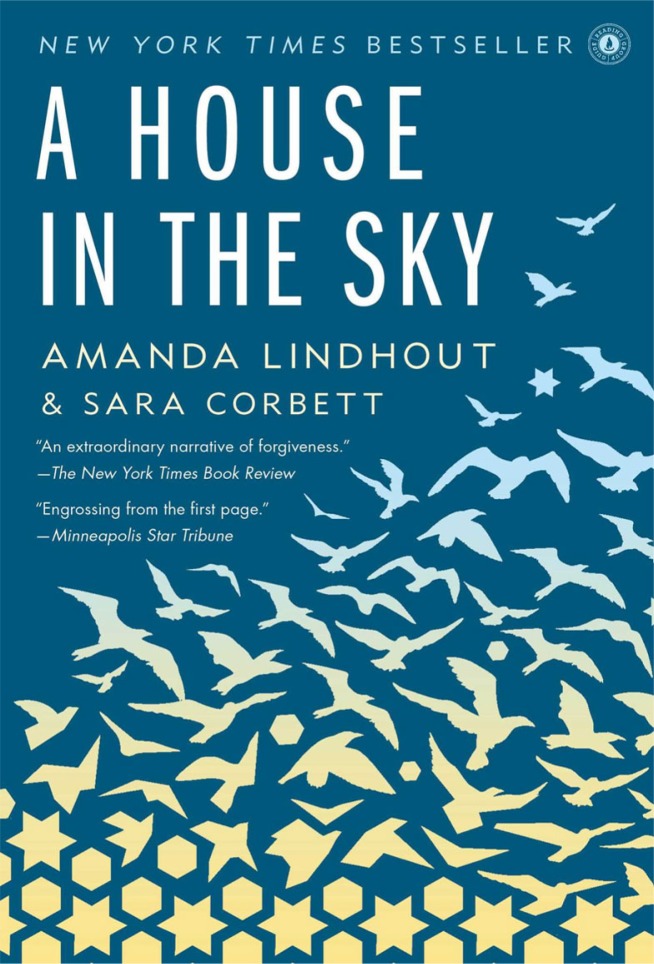


Review of *A House in the Sky* by Amanda Lindhout and Sara Corbett (2014). New York, United States: Scribner.400 pp. ISBN 978-1-45165613, 16 USD.

“And hope, we all agreed, is the best thing in the world” concludes *A House in the Sky*, the riveting chronicle of Amanda Lindhout's journey from her childhood home in Alberta, Canada, to her kidnapping and torture in Somalia to her founding the Global Enrichment Foundation. This conclusion is fitting as in *A House in the Sky*, Lindhout and Corbett give their readers the gift of hope.

I started *A House in the Sky* on the train to the 29th Annual Meeting of the International Society of Traumatic Stress Studies. To be frank, I read it out of a sense of obligation. As I have confessed previously, I avoid reading anything about trauma or posttraumatic stress disorder (PTSD) when I am not working. However, I was president of ISTSS and had asked Amanda to be our opening speaker. Amanda had donated 250 copies of her book to be given out at her talk.

Once I started reading *A House in the Sky*, I could not stop. I knew Amanda's story. It had been covered widely in the media. The New York Times Sunday Magazine published an excerpt of her book. Despite this, from the opening sentence of the Prologue, “We named the houses they put us in,” I was riveted. My days, prior to the opening of the annual meeting of ISTSS, were booked solid with committee, board meetings, and various presidential duties. I read *A House in the Sky* walking down hotel corridors between meetings, while brushing my teeth, and late into the night, breaking one of my cardinal rules about no trauma reading in bed.

Why did I find *A House in the Sky* so compelling?

The reason was Amanda's unwavering faith that there is something better. In the first chapter, *My World*, the reader gets to know Amanda as a young girl. She hunts through garbage bins, scrounging for money by recycling bottles to pay for her beloved *National Geographic* magazines. She uses her imagination to escape the violence in her home, “My mind swept from beneath the bedsheets, up the stairs and far away, out over the silky deserts and foaming seawaters. … My world, I was pretty certain, was elsewhere.” As a reader, I wanted nothing more than to join Amanda on her journey to find her world.

Lindhout and Corbett's skillful description of Amanda's captivity enables the reader to experience the events with Amanda rather than objectifying her. When writing about trauma, there is always a risk of making the details of the event more vivid and engaging than their impact on the survivor. The description of the trauma becomes voyeuristic entertainment for some, triggering for others. Amanda experienced extreme violence, including gang rape, at the hands of her kidnappers in Somalia. Lindhout and Corbett do not gloss over these events:

Then he was on top of me, and I was hating him with every molecule in my body. I wanted him to die. I put my hands against his chest to create a sort of barrier between us. Something in me howled … and something happened. … I wasn't in my body anymore ….

This narrative choice makes *A House in the Sky* more, rather than less powerful.

Rather than focus on the graphic details of what was done to her, Lindhout and Corbett focus on Amanda's experience of these events and how she transforms them:

Images ran past me, scenes from stories Abdullah had told me months earlier. His life was abruptly on display. I saw him as a young boy, running from an explosion … For one split second, I knew his suffering.

From that moment, Amanda works diligently to grow the seeds of compassion she feels for her captors. When freed, she begins the Global Enrichment Foundation, a non-profit foundation whose mission is to provide education for Somali women. An inspiring transformation.

But even more inspiring for me is Amanda's commitment to her recovery. She touches on this in *A House in the Sky* but describes it in more detail in her article in this issue. She continues to suffer from PTSD symptoms and the physical health consequences of her captivity. Yet, despite this, as she told me, when I called to ask her to give the ISTSS plenary, “Every day I choose healing and forgiveness.” In her article in this issue she says:

I still have my struggles—I am still afraid of the dark, loud noises make me jump and when a man gets too close, I want to run. But I do see that healing is taking place and I am learning to live with my trauma. I'm coming around to the idea that, while the kidnapping will always be a part of my life story and who I am, the trauma of it does not have to control me.

And with that statement, Amanda's story of her recovery, like *A House in the Sky*, provides hope for those of us who work in the trauma field and for the survivors we serve.

